# Effect of an Early-Age Exposure on the Degradation Mechanisms of Cement Paste under External Sulfate Attack

**DOI:** 10.3390/ma16176013

**Published:** 2023-09-01

**Authors:** Othman Omikrine Metalssi, Rim Ragoug, Fabien Barberon, Jean-Baptiste d’Espinose de Lacaillerie, Nicolas Roussel, Loïc Divet, Jean-Michel Torrenti

**Affiliations:** 1 Laboratoire Matériaux pour une Construction Durable (UMR MCD), University Gustave Eiffel, Cerema, F-77454 Marne-la-Vallée, France; 2 Organisation Professionnel Prévention Bâtiment Travaux Public, 25 Avenue General LECLERC, 92600 Boulogne Billancourt, France; rim.touhami@oppbtp.fr; 3 EQIOM, 49 Avenue Georges Pompidou, 92300 Levallois-Perret, France; fabien.barberon@eqiom.com; 4 Soft Matter Science and Engineering, ESPCI Paris, UMR CNRS 7615, Sorbonne Université, 75005 Paris, France; jean-baptiste.despinose@espci.fr; 5 Laboratoire Navier, Université Gustave Eiffel, CPDM, F-77454 Marne-la-Vallée, France; nicolas.roussel@univ-eiffel.fr; 6 Civil Engineering Department, Sherbrooke University, 2500 Boulevard de l’Université, Sherbrooke, QC J1K 2R1, Canada; loic.divet@outlook.com

**Keywords:** external sulfate attack, physicochemical behavior, early-age effect, sulfate ingress

## Abstract

Among the most significant causes of concrete degradation is ESA (external sulfate attack). The majority of studies are currently conducted on samples that have been saturated and matured. Concrete structures, however, are exposed to the environment once the formwork has been removed. The purpose of this study is to determine what effects early exposure to external sulfates may have on degradation mechanisms. Microstructure, physical, and chemical behavior are monitored using a variety of experimental techniques, including NMR (^27^Al and ^29^Si), ICP, XRD, MIP, and SEM. Based on expansion measurements, mature Portland cement paste, unlike the early-age case, degraded rapidly due to the presence of compressed ettringite and gypsum, highlighted by SEM analysis. During ESA, sulfate ions diffuse through the cement matrix and are bound by chemical agents. Chemical analyses indicate that the chemical mechanism varies with the duration of curing. At an early age, external sulfates and aluminates are the most important reagents. For matured cases, these reagents include external sulfates, calcium derived from CH dissolution, and aluminates derived from the total dissolution of AFm.

## 1. Introduction

Civil engineers continue to struggle with the long-term behavior of concrete structures. In most cases, when the formwork is removed from concrete structures, a variety of physicochemical aggressions occur. As a consequence of this early-age exposure, the future transport properties of the structural element are influenced, which are important parameters determining the durability of the material. Transport properties affect the material’s ability to sustain the degradation mechanisms resulting from the penetration in the cementitious matrix’s inner porous microstructure of deleterious ions and aggressive elements (CO_2_, sulfates, and chloride ions, etc.) by the external environment [1,2,3,4,5,6,7]. Early-age exposure can also influence the mechanisms of internal reactions, such as the well-known alkali–aggregate reaction (AAR) and delayed ettringite formation (DEF) [8,9,10]. Two phenomena, the alkali’s content of the concrete and the temperature reached during the concrete hardening and relative humidity, influence the extent of degradation at an early age. Alkalis are primarily obtained from cement, although some may also be released from aggregates. The presence of a high-alkali content at an early age will cause sulfates to be released, which provide the reagents needed to form ettringite. In terms of temperature, DEF effects are related to the material’s thermal history. Most scientists believe that an increase in temperature or duration of heating during hydration at an early age increases the kinetic and ultimate values of expansion. The expansion of the material increases with the increase in humidity, which is higher during the first phase of hydration. Ettringite also contains water as one of its constituents. Since most standards and laboratory tests pertaining to durability are conducted on mortar or concrete samples that have been cured for 28 or 91 days, the effect of early-age exposure is still unclear and requires more laboratory investigations.

External sulfate attack (ESA), considered to be the second leading cause of concrete structure degradation after corrosion, is one of the most hazardous phenomena that can cause concrete structure degradation. ESA is also one of the most cited causes of concrete structure deterioration. In general, this phenomenon can be attributed to the formation of ettringite and gypsum, under some conditions, when sulfate ions and alumina constituents react in cement [11,12,13,14,15,16,17,18,19,20]. There is general agreement that the precipitation of these newly formed elements in the cement matrix leads to considerable expansion and cracking in structures. This can cause surface spalling accompanied by an overall loss of structural integrity highlighted by reductions in stiffness and strength [14,21,22].

ESA is a complex multiscale and multiphysics phenomenon that involves physical, chemical, and mechanical interactions [14,20,23,24,25,26,27,28]. Although these interactions are relatively well understood individually, it remains difficult to predict how they pair together and the overall phenomena remains unclear and highly controversial, even for ordinary Portland cement, for which, unlike other cements, substantial results have been obtained [29]. The complexity of the problem begins with the variety of sulfates that can damage concrete (calcium, sodium, and magnesium sulfate). In the case of sodium sulfate, one school of thought holds that the mechanism of ESA begins with gypsum formation before expansive ettringite formation [30], while another school of thought suggests the opposite process [31]. Furthermore, the mechanism of the ESA depends on the amount of sulfate present in the external solution. Based on Biczok’s results [32], in low-concentration sulfate environments, ettringite forms as the predominant product, while in high-concentration environments, gypsum forms as the dominant product.

Although many publications and research studies have been conducted about the ESA mechanisms [33], there remain several unanswered questions and, in particular, the early-age behavior of structures exposed to ESA still requires further study. This early age has a significant impact on the long-term service life of concrete structures, as constituent proportions of concrete and its microstructure undergo continuous and rapid changes during this period. It may be difficult to achieve high-quality concrete when the early-age properties of concrete are not sufficiently taken into account. Material can undergo internal and/or external deformations that can result in cracking and the loss of its mechanical properties. As a result, the material may become weaker and more susceptible to penetration by aggressive agents, such as ESA. Therefore, at an early age, the coupling between the hydration process and mechanical properties is more critical than in mature concrete. It is also possible for poorly cured concrete to seriously compromise its mechanical performance and long-term durability. The proper curing of concrete after placement is essential to maintain satisfactory moisture content and the necessary temperature during this early age of concrete’s development in order to achieve its intended properties.

This study examines the kinetics of sulfate ingress and its impact on the microstructure and degradation mechanisms of cement paste at an early age. Both at early ages and after one year of curing in water, the sulfate concentration profiles were determined by ICP (inductively coupled plasma analysis). SEM analysis was used in conjunction with these measurements to investigate the microstructural changes. Furthermore, other investigations were conducted using nuclear magnetic resonance (NMR), ^27^Al and ^29^Si, and X-ray diffraction (XRD) to analyze the chemical composition of newly formed elements during ESA. Lastly, a visual comparison of the degradation of the samples and the expansion measurements for the two different curing conditions were conducted.

## 2. Materials and Methods

### 2.1. Formulations and Exposure Conditions

For the purpose of this study, Portland cement (CEM I) with a chemical composition as shown in Table 1 was used for casting cement paste specimens. This composition was obtained by a combination of inductively coupled plasma analysis (ICP) and thermogravimetric analysis (TGA). According to Bogue’s approach, the clinker phase contents are as follows: C_3_S = 65.2, C_2_S = 8.8, C_3_A = 7.9, and C_4_AF = 8.9. According to these values, there is a significant amount of aluminate in the form of C_3_A and C_4_AF, which makes it likely that secondary ettringite will be formed after a reaction with a large amount of sulfate ions.

**Figure 1:**
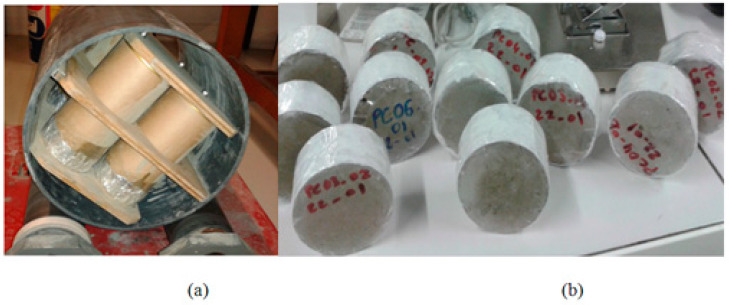
A device used during the cement paste’s hardening (**a**), specimens used for the study (**b**).

**Figure 2:**
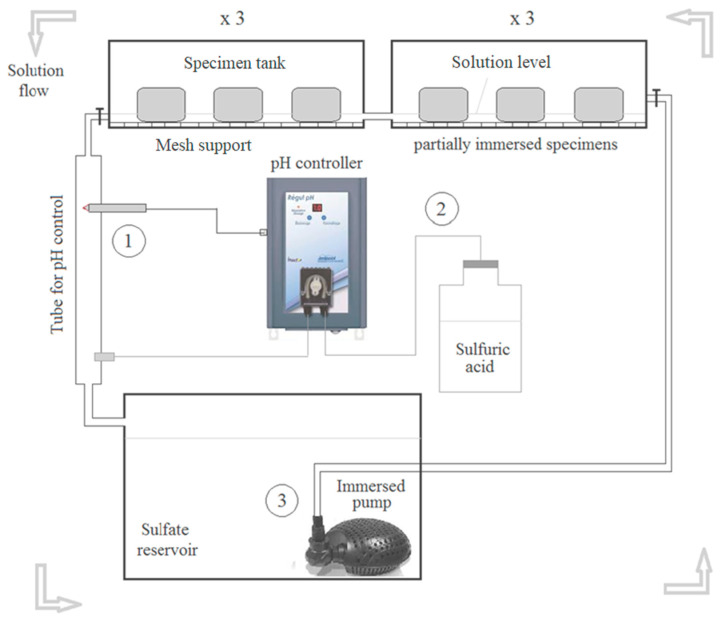
A pH-control scheme for accelerated tests at ESA.

For the preparation of the specimens, cylindrical samples with a 10 cm diameter and 15 cm length were cast with cement paste and labeled as the OPC samples. To maximize porosity and enhance sulfate ingress, a high water-to-cement mass ratio (W/C) of 0.6 was selected. As part of the setting procedure, the cement paste was prevented from bleeding with the use of a sample slow-rotation device. This may ensure the homogeneity of the samples (Figure 1a). Once the specimens had been removed from their molds for 24 h, they were cut into 5 cm long cylinders. One third of the samples were then kept directly in sulfate solution (early-age exposure case) and the remaining two thirds were cured in non-renewed water for one year (matured case). One of these two thirds was further exposed to external sulfate solution after a long curing period of one year (matured exposure case), under the same conditions as the early-age case samples. Finally, as a reference case, the last third of the samples was cured in tap water. All cylindrical samples were coated with epoxy resin on their lengths, but not on their bases in order to ensure unidirectional diffusion of sulfate ions (Figure 1b). Hence, there was only one base of the specimen that was in contact with the test solution (semi-immersion) (Figure 2), which was prepared by dissolving 15 g/L of sodium sulfate in deionized water (10 g/L of sodium sulfate). There was a 25 cm^2^/L ratio between the surface area of the sample and the volume of the solution. As discussed in more detail below, a pH regulator was used to maintain the pH of the sulfate solution at (8 + 0.1).

Companion cylindrical specimens with a 10 cm diameter and 15 cm length were used to measure the axial expansion during the degradation process for the early-age and matured cases. After demolding, three specimens for each exposure condition were equipped with stainless steel pins to ensure that the axial dimension was monitored. A digital extensometer and a steel reference length bar were used for these measurements.

### 2.2. Accelerated Test for ESA

To study the resistance of cementitious materials to ESA, using a constant pH = 8 ± 0.1 is preferable to field conditions (e.g., seawater) [34]. Due to the constant sulfate concentration and pH of our proposed accelerated test method, the experimental conditions are more representative of field conditions. Several studies have used high sulfate amounts in their experiments [35,36,37]. Figure 2 shows a schematic of the test apparatus.

In this simplified device, sulfate solution with a concentration of 15 g/L is contained in one large tank that serves as a reservoir for approximately 60 L of solution. The sulfate solution was circulated through six smaller tanks containing 10 L each with the aid of an immersion pump with a maximum flow rate of 750 L per hour (Figure 2). Before flowing back into the sulfate reservoir at the output of these tanks, a controller continuously monitors the pH of the solution (process 1). The pH of the sodium sulfate solution was determined through continuous titration with sulfuric acid H_2_SO_4_ (0.05%) (process 2). At the exit of the tanks containing the specimens, a pH electrode is used to continuously measure the pH of the solution. As part of this system, an actuator injects diluted sulfuric acid into the solution whenever the pH level increases due to leaching, so that it returns to the target pH level. The added sulfuric acid is well mixed since the sulfate solution is constantly flowing between the sulfate reservoir and the specimen tanks (process 3).

In each of the six tanks, semi-immersed samples of similar chemistry were arranged on mesh supports so that the cement paste and sulfate ions were exposed to the ESA to maximize the chance of the cement paste reacting with it. Only 1 cm thickness of each specimen was immersed in the sulfate solution, while the other 4 cm of thickness remained out of solution. Sulfate concentrations were minimized by adjusting the volume ratio between the specimens and sulfate solution. This parameter was kept approximately constant by weekly solution renewals. Sulfate ions were maintained almost constant in solution over time due to the use of sulfuric acid in the titration. The same device was used for expansion measurements in sulfate solutions.

### 2.3. Experimental Techniques for Investigation

The kinetics and effects of sulfate ingress in ordinary cement pastes’ porous media were examined using a variety of test methods. All physicochemical investigation methods were applied to a powder ground from the surface of the test samples every 1 mm. As part of this process, Germann Instruments’ Profile Grinder tool (Denmark) is used to reduce concrete, mortar, or cement paste into fine powder by precision grinding to small depth increments of 0.5 to 2 mm. As a result, it is possible to determine with high precision the profiles of ionic contents and chemical elements at each depth. In our case, the ground area has a diameter of 73 mm (which justifies the selection of cylinder samples with a diameter of 100 mm). This diameter makes it possible to obtain about 9 g of powder for 1 mm depth. The average fineness of the powder is 315 μm. Between each depth, the powder is carefully picked up and the surface is vacuumed to prevent contamination.

The sulfate content of the samples was determined using inductively coupled plasma atomic emission spectroscopy (ICP). Sulfate concentration is calculated from the total sulfur concentration obtained during this experiment. The exposed surface layer of the samples to a depth of 1.5 cm was ground. The resulting powders had an average size of 300 µm. An acid attack ionized all sulfates in this powder. An ICP-AES measurement was then conducted on the resulting solution. The experimental procedure used in this part of the study which details the procedure for preparing samples to measure the concentration of SO_3_ is explained in [23]. Additionally, Mercury Intrusion Porosimetry (MIP) measurements were conducted to examine the effect of ESA on pore size distribution.

Additionally, the spatial distribution of different cement paste phases was also determined by scanning electron microscopy (SEM, Quanta 400 from FEI Company, Hillsboro, OR, USA). Despite the fact that this technique provides only qualitative information, SEM images can be used to distinguish between phases that are well crystallized and do not harm the microstructure, such as compressed ettringite, from phases that are crystallizing in well-distributed free volumes.

Furthermore, a METTLER TOLEDO TGA/DSC1 was used for thermogravimetric analysis (TGA) coupled with differential thermal analysis (DTA). During the ESA, these methods were used to quantitatively characterize cement pastes. Using these techniques, this study could explain the mechanism by which AFt is formed from the consumption of other cement hydrates.

In addition, X-ray diffraction (XRD) was carried out in this research. XRD can be used to identify well-crystallized hydrates in cement paste, such as portlandite or ettringite. As a result, a mineralogical profile is drawn and different types of mineralogical phases are identified according to the type of exposure. The results obtained by XRD were also confirmed by ^27^Al and ^29^Si NMR using an Avance III Bruker spectrometer (Martin Dracinsky Group, Czech Republic) ^27^Al MAS NMR was performed to calculate the amount of different hydrated alumina phases (AFm and AFt) within the cement. ^29^Si NMR was used to investigate the C-S-H structure changes due to sulfate attack. ^27^Al MAS NMR one-pulse spectra were obtained by modeling the Czjzeck lineshape using the DMFIT software [38] following the procedure described in [39]. ^29^Si NMR one-pulse spectra were obtained by using a Gaussian lineshape with the DMFIT software, and the mean chain length (MCL) calculated according to [40].

As a final step, macroscopic observations were conducted on samples exposed to ESA for various periods of time.

## 3. Results 

### 3.1. Expansion Measurements

**Figure 3:**
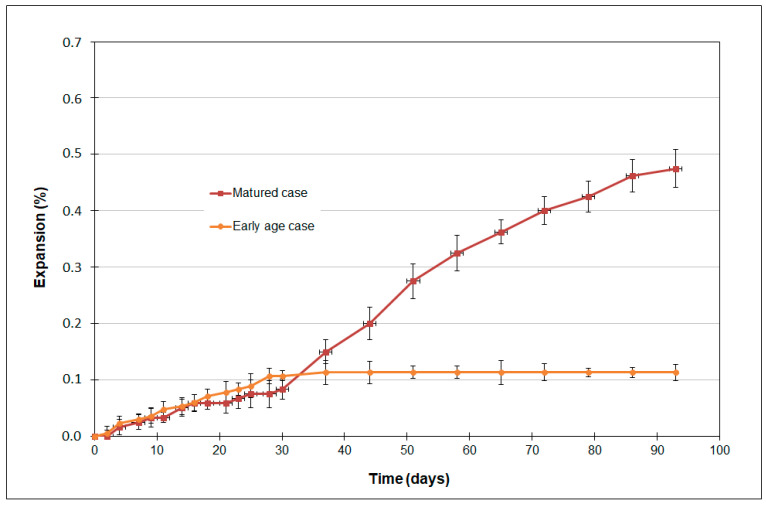
Expansion variation for the early- and mature-exposure cases.

Figure 3 shows the average expansions and standard deviations obtained from axial length measurements on three 10 cm × 15 cm cylindrical samples. These curves are for early- and mature-exposure cases. A significant expansion is observed at the end of the test for the matured exposure case (about 0.47%), with the speed divided into two parts: a latent period up to about 30 days in which expansion remains weak, followed by a period in which the expansion speed increases rapidly and remains high until the end of the test (around three months). This change in regime was a result of changes in the microstructure of the samples. Indeed, the acceleration of the expansion is due to the clogging of the porosity by the progressive formation of secondary ettringite and gypsum. It has been observed in contrast that, in the early-age exposure case, the sample exhibited modest axial expansion (up to 0.12%), followed by a gradual slowdown over time. These contrasted results could only be due to variation in the microstructure since the bulk quantities of reagents present in both exposure conditions were identical. These results are in concordance with those of [13]. The details of this will be discussed in the following sections.

### 3.2. Sulfates Profiles

**Figure 4:**
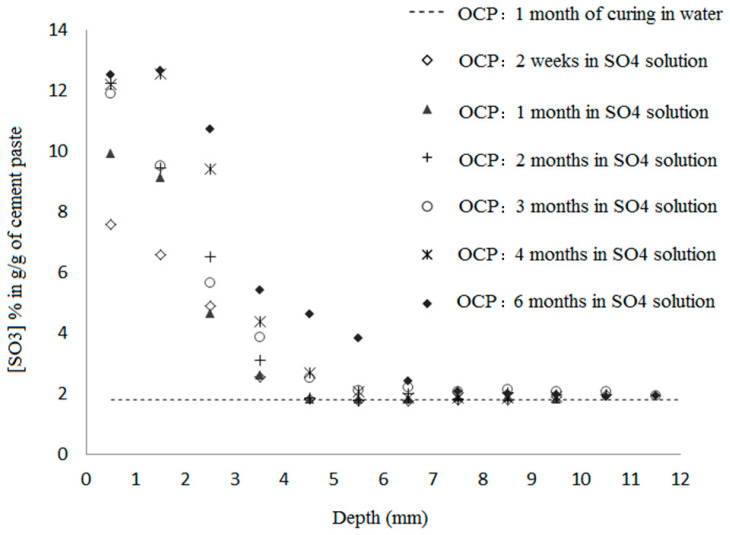
Profiles of sulfate at early age measured by ICP, for different exposure duration to the ESA.

Figure 4 illustrates the evolution of sulfate content in OCP samples versus depth under early-age exposure. The curves are divided into two parts: one displaying the ingress of sulfate into the cement, and the second maintaining the average sulfate concentration of the reference cement without being exposed to sulfate solution.

As the exposure time increases, the sulfate content of the cement increases until it stabilizes at approximately 13% g/g near the surface. This value differs considerably from what might be expected from a simple equilibrium between the internal pore solution and the external sulfate solution (about 0.2% g/g of cement). Therefore, the pore solution is dominated by physical or chemically bound sulfates, which explains the decrease in sulfate content with depth. Furthermore, the extremely high ratio of solid or adsorbed sulfates in the cement matrix required to free sulfates in solution, estimated to be of the order of 60, suggests that the fixation process involves an extremely high level of energy, assuming the free sulfates’ concentration in the pore solution is of the same order as the external solution sulfate concentration. Thus, sulfate physical adsorption alone is not sufficient to achieve this binding, which is mostly chemical in nature. As stated in [41], the quantity of adsorbed sulfate on C-S-H was estimated to be 1% g/g, which is considerably lower than the average sulfate content reported here of 13% g/g. As the exposure time increases, the penetration depth increases slightly as well. As a result of two weeks’ exposure to sulfate solution, it measures approximately 4.5 mm, and at six months, it measures approximately 7.5 mm.

**Figure 5:**
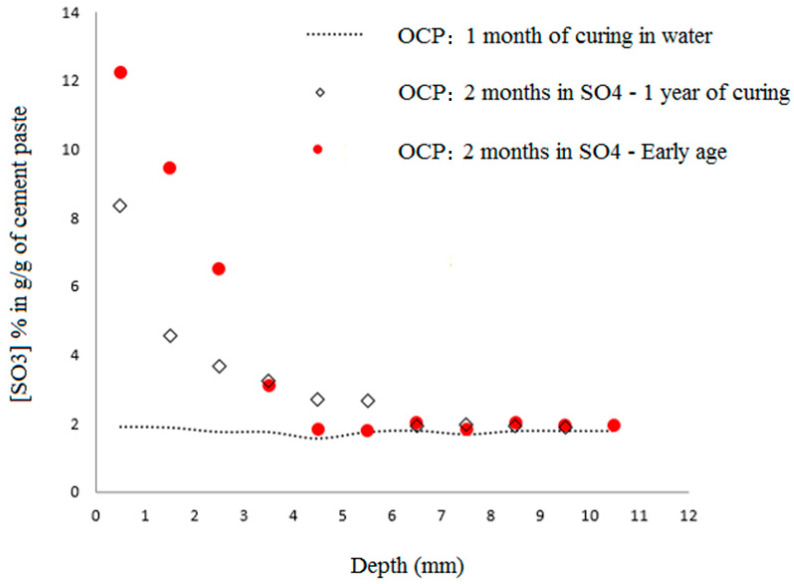
Comparison of the sulfate profiles between the two exposure conditions: early-age and mature cases.

A further illustration of this development is shown in Figure 5. This illustrates the evolution of sulfate content in OCP materials over time for both early and matured exposure modes to sulfate solutions. Despite similar sulfate profiles in both cases after two months of sulfate exposure, there is a difference in the maximum sulfate content attained. Early-age exposure to the first three millimeters results in a higher maximum value. It is a result of the high porosity and permeability of the material at this early stage. In an area near the propagation front, the difference is negligible above this depth. The results of these experiments indicate that although sulfate ions are initially trapped in the porous medium via a diffusion process, they are physically and chemically trapped in the microstructure of the paste at an important kinetic rate. In addition, capillary suction may also contribute to sulfate ingress, especially when samples are exposed at an early age.

The maximum sulfate penetration depth also increases in matured samples. As a result of the samples’ exposed surface having developed some cracks after two months of exposure, this may be the cause of the results. Indeed, after long curing in water, the material becomes rigid and little ettringite formation or other expansive products can cause cracking. The situation is different for materials that are still in the early stages of development, when the matrix is not yet rigid, and expansive products are able to find space to form without causing internal pressures and, consequently, without cracking. Sulfate ions and other deleterious ions may be able to pass through these cracks.

### 3.3. Chemical Mechanisms

**Figure 6:**
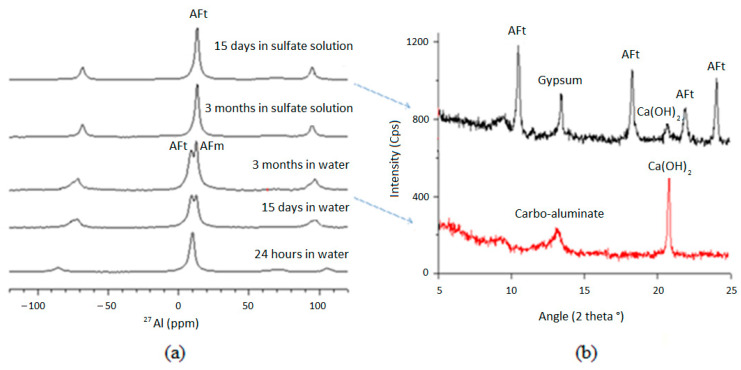
Determination of phases newly formed by ^27^Al MAS NMR (**a**) and XRD (**b**) after exposure of OCP samples to the sulfate solution.

Sulfate ions will certainly affect the chemical equilibrium of the cement paste as they are absorbed by the cement matrix. In cement, sulfate ions form secondary ettringite and gypsum when they react with calcium, aluminum, and water. On the other hand, when cement samples are leached in a sulfate solution, the pH difference between that solution and the pore solution of the samples results in an increase in the calcium content of the pore solution which in turn favors AFt formation [23,24,25,26,27]. This exemplifies how ESA and leaching are strongly coupled and compete with one another. According to Figure 6, the phase assembly in the cement paste underwent a change after being exposed to sulfate solution. A combination of XRD and ^27^Al NMR analyses indicates that, on the one hand, secondary ettringite was formed as a result of reactions between portlandite and sulfate ions; and on the other hand, that AFm transformed to AFt. In addition, the XRD analysis indicates that there is a considerable amount of gypsum on the exposed surface of the samples, as this area is almost in equilibrium with the pH of the sulfate solution (pH = 8). This pH value results in both ettringite and gypsum precipitation. Moreover, the high rate of anhydrous compounds at an early age allows for the production of more secondary ettringite. Indeed, the aluminates present in C_3_A and C_4_AF react directly with the sulfate ions to produce ettringite. In the matured case, this mechanism does not exist. All anhydrous compounds have already been consumed during the hydration process.

**Figure 7:**
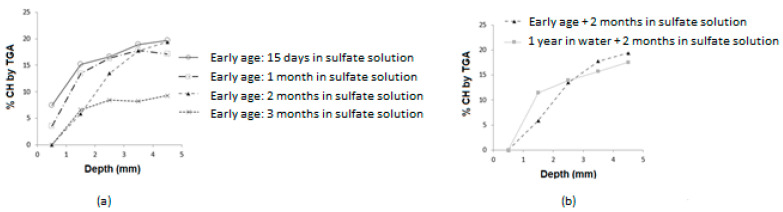
Quantification of CH content by TGA: (**a**) early-age case, (**b**) comparison early-age/matured cases.

Figure 7 illustrates the results of TGA. These results are consistent with those obtained by XRD. In fact, portlandite is the first hydrate to be consumed by sulfate ions that penetrate the first millimeters of the surface of the samples (Figure 7a). It is important to note, however, that the consumption does not appear to be the same for both cure conditions. As the material is completely hydrated after one year of curing in water, the amount of portlandite is much greater in the mature case (Figure 7b). In the presence of sulfate ions in the cementitious matrix, they thermodynamically destabilize the hydrates, particularly portlandite, resulting in its dissolution. Calcium from this hydrate reacts with sulfates to form gypsum, especially at neutral pH levels. Accordingly, portlandite dissolves more rapidly when subjected to a sulfate attack than when not exposed. In the process, gypsum replaces calcium hydrates, which results in a decrease in portlandite content.

**Figure 8:**
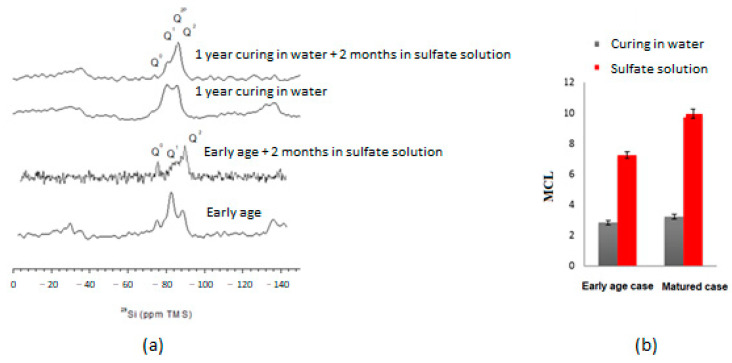
Changes of C-S-H structure investigated by 29Si NMR: (**a**) spectrum, (**b**) mean chain lengths in C-S-H.

Figure 8a shows the ^29^Si MAS NMR spectra for both early and mature exposures. The peak of the tetrahedral at the end of the C-S-H chains, Q^1^, has decreased while that of the tetrahedral in the middle of the chains, Q^2^, has increased. This means that the C-S-H mean chain length (MCL) increased and that the C-S-H structure changed. The MCL of the C-S-H chains increased to about 7.5 in the early-age exposure case and to about 10 in the matured exposure case (Figure 8b). During C-S-H decalcification (C/S ratio decrease), it is well known that the MCL in C-S-H increases [40].

Once the calcium has been consumed by dissolving the portlandite, the C-S-H are decalcified, increasing the calcium content in the interstitial solution. The calcium then reacts with excess sulfates to produce gypsum and secondary ettringite. In the mature case, calcium content is greater, suggesting that the development of ESA is more dependent upon the presence of a high-hydrate content.

**Figure 9:**
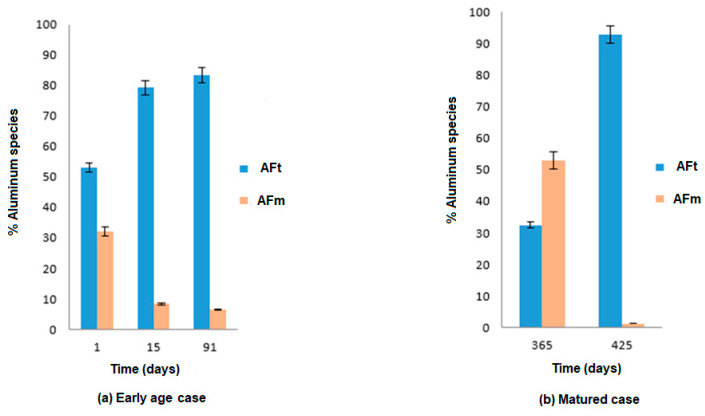
Quantification of Aft and AFm content for the two curing durations.

There is a drastic difference in the AFt/AFm balance between the two exposure cases (Figure 9). In the case of early-age exposure, this ratio is higher. In Figure 9a, the secondary ettringite is partly produced as a result of the progressive dissolution of AFm. The mature-exposure case (Figure 9b) results in the complete consumption of AFm. These results suggest that ESA develops differently depending on the length of time the curing process is carried out prior to exposure. In sum, at an early age, AFt is formed through the reaction of the sulfates with part of the AFm and also through the reaction of the aluminates produced by the anhydrous compounds C_3_A and C_4_AF. When a mature paste is exposed, this formation of AFt is mainly due to the total consumption of AFm and to the contribution of calcium coming from CH dissolution and C-S-H decalcification.

### 3.4. Microstructure Changes

**Figure 10:**
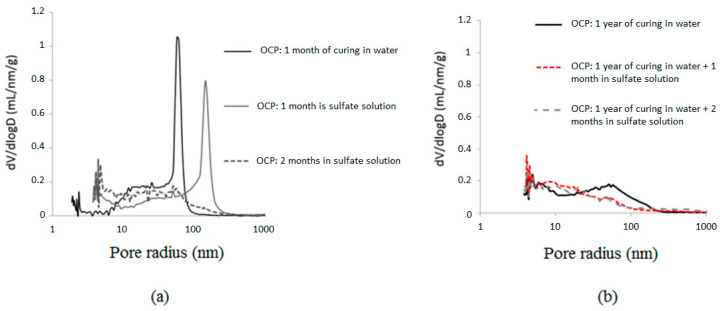
Evolution of the pore size distribution of OCP samples during ESA: (**a**) early-age exposure, (**b**) matured cement pastes.

Figure 10 compares the pore size distributions between cases with and without exposure to ESA for OCP samples at different exposure times. After one month of exposure to ESA, the effect of leaching is highlighted. The distribution of pore size after 2 months of ESA, however, is refined and tends towards the range from 10 nm to 100 nm (Figure 10a). Clearly, at an early age, hydration, leaching, and precipitation of sulfated species were competing processes, making it difficult to distinguish between them. Moreover, in the case of matured cement pastes (Figure 10b), the ESA appears to be more damaging for cementitious materials since the pore size distribution still tends to a smaller range (of the order of 10 nm), despite the quasi-complete hydration of the material, which suggests that the precipitation of the AFt and the gypsum occurs within a much smaller porosity than when the material is exposed at an early age.

**Figure 11:**
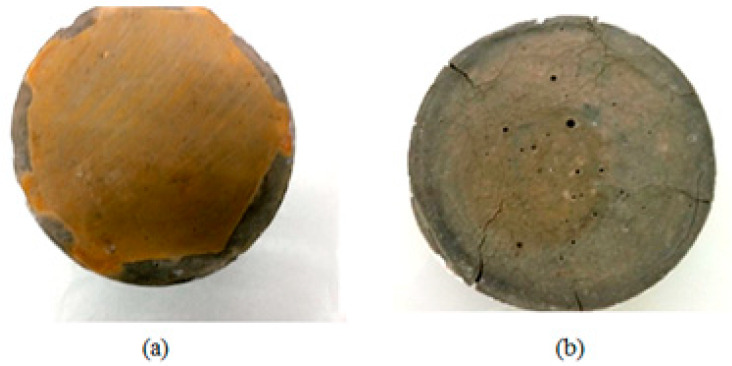
Observed damage of samples after two months of exposure to sulfate solution: (**a**) early-age exposure, (**b**) matured cement pastes.

A significant expansion and cracking of concrete structures is generally attributed to secondary ettringite and gypsum as a result of sulfate attack on hydrated alumina phases in the cement matrix. Visual examinations of samples exposed to sulfate solution after different exposure periods were conducted to confirm the role of sulfates in cementitious materials’ damage. Figure 11 illustrates a comparison of samples at an early age (Figure 11a) and matured age (Figure 11b) after two months of exposure to sulfate solution. Early exposure did not show apparent degradation or cracking. After one year of contact with sodium sulfate solution, these observations remain valid. After just two months of exposure to sulfate, the mature samples have developed some cracks.

**Figure 12:**
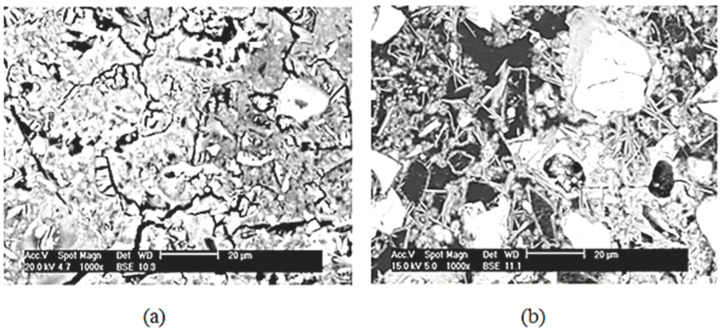
SEM images on OCP samples after two months of ESA: (**a**) compressed ettringite in matured samples, (**b**) ettringite in early-age samples.

Accordingly, it appears that AFt phases crystallize more readily in smaller pores and after a more advanced hydration reaction (saturated pores in matured samples) thereby resulting in greater cement paste risks (ettringite has approximately 2.28 times the volume of AFt phases; 707 cm^3^ versus 309 cm^3^). Additionally, mature samples are sufficiently rich in portlandite and C-S-H to precipitate gypsum upon dissolution. The situation is different if the exposure occurs at an early age. Moreover, gypsum has a 2.25 times greater molar volume than portlandite (74 cm^3^ compared with 33 cm^3^ per mole). As a result, the newly formed elements after an ESA (AFt and/or gypsum) could increase the expansion of cement paste samples when confined in porosity. Furthermore, SEM analysis of samples exposed to sulfate for early-age and mature cases has confirmed this observation. Based on Figure 12, the ettringite precipitates in confined spaces in mature samples (Figure 12a), confirming that the samples have expanded and cracked, while at an early age, the ettringite has longer spaces (Figure 12b).

## 4. Discussions

Experiments conducted within the framework of this study demonstrated firstly that the ESA is characterized by two major processes, a physical process of ionic transport and a chemical process of interaction with the microstructure. The first process is characterized by both a transport of sulfate ions towards the cementitious matrix and a leaching of calcium ions (resulting in the leaching of hydroxides from the portlandite) in the external solution. ICP was used to measure sulfate concentration profiles, which demonstrated that ESA is driven by diffusion and that chemical interactions (the second process) between hydrates and external sulfate supplies are more rapid than diffusion kinetics. In addition, sulfate ions have been demonstrated to self-slow during propagation. In the measured profiles, the sulfate ion migration front is rapidly stabilized followed by an accumulation of sulfate ions at the surface. Based on ATG/AD, DRX, and NMR analyses, this accumulation was explained by the fixation of sulfur ions by the precipitation of AFt as long as aluminum and calcium reserves were present, as well as the coexistence of gypsum and ettringite at times on the surface (especially in mature Portland cement pastes). 

The effect of curing time on the degradation of the cementitious materials in contact with sodium sulfate solution was demonstrated in several steps. The first method is visual observations which show that Portland cement pastes resist ASE well when exposed early to sulfates (no degradation is observed after one year). The mature material, however, deteriorates rapidly and suddenly on the surface and then deeper towards the core after only two months of sulfate exposure. Hence, expansion is directly affected by porosity and pore size distribution. According to SEM visualization and MIP tests, samples for the early-age case have a greater amount of porosity, allowing them to accommodate expansive phases without sustaining damage. A long-term curing in water results in a rapid and sudden filling of the porosity, resulting in a sudden change in the sharpness of expansion curves for mature samples due to the fine microstructure and rigidity of the material. 

In cement paste, the pore size distribution of capillary pores and hydrate pores has a significant effect on the transfer properties, particularly when the pores are interconnected. This observation is supported by the profiles of sulfate penetration that are much greater in the case of coarse porosity (early age). It should be noted that the chemical aspects of ESA are almost insensitive to these transport properties (especially in the case of an early-age exposure), but rather to the chemical reactions taking place within the material. These reactions are dependent on portlandite, the AFm and AFt phases, as well as the aluminum incorporated into the C-S-H prior to exposure to the sodium sulfate solution.

On the other hand, the quantification of precipitated ettringite in the two exposure cases demonstrated that degradation is not directly related to changes in molar volume. According to the hydrate distribution at an early age, ettringite was more abundant than in mature paste after two months of ASE. Nevertheless, these abundant precipitations were not the cause of the material’s damage. In order to explain the cracks induced by tensile stresses, the crystallization pressure theory under supersaturation conditions was adopted in this study.

Based on the curing time of the material, the physical chemistry of ASE differs. As previously explained, this refers to chemical reactions rather than sulfate ions’ transport, which is almost insensitive to curing duration. AFm/AFt ratios are very low when OPC is exposed at an early age. The investigation techniques used in this study indicated that ettringite continues to form and crystallize in a wide and evolving porosity with a hydration phenomenon occurring in the presence of sulfate, without causing damage to the matrix during the crystallization process. Conversely, when exposed to sulfates after 1 year of water hydration, the AFm/AFt ratio is significantly higher and portlandite is more available. Ettringite precipitates by dissolving CH and AFm and by decalcifying C-S-H. Gypsum is detectable by scanning electron microscopy and XRD as long as calcium and sulfate ion concentrations allow. These hydrates crystallize in a much more confined porous environment and a much more deleterious effect can be expected.

Based on this difference, it is evident that when concrete structures are exposed to sulfate-rich environments, such as marine environments or sulfate-rich soils, the curing time of these structures should be reduced. The structures would have a greater longevity if they were directly cured in a sulfate-rich environment. As previously explained, the material exposed directly to sulfate ions does not show any signs of degradation or cracking.

**Figure 13:**
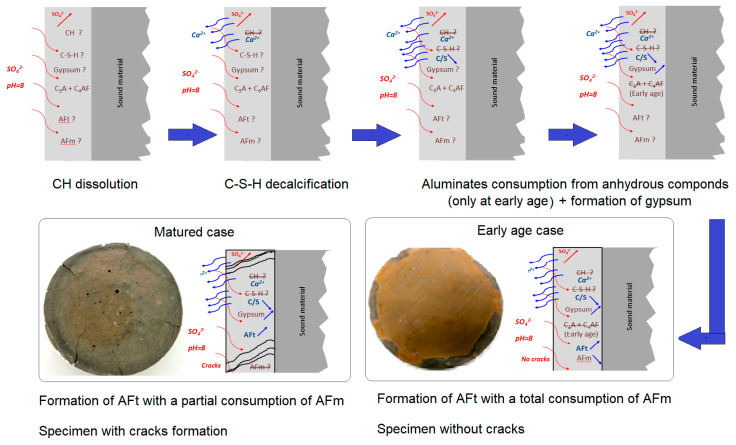
Mechanisms of ESA development for both early and mature curing durations.

Therefore, this work has provided insight into the negative effects of long time curing in the case of Portland cement. However, these results are only valid for the CEM I used in this study. The cement, which is very rich in C_3_A and C_4_AF, was chosen despite its potential reactivity with sulfate ions. In Figure 13, the mechanisms of ESA development are shown step by step for both early and mature curing durations. Sulfate ions penetrate the cementitious matrix where they react directly with calcium ions resulting from the dissolution of the portlandite to produce gypsum and AFt. After these calcium ions have been consumed, the C-S-H decalcifies, thereby providing an additional quantity of calcium needed to form gypsum and AFt. This resulted in an increase in the C/S ratio. Another part of the sulfate ions reacts directly with the sources of aluminates (AFm, C3A, and C4AF) to form AFt. According to the previous results, curing duration affects the chemical mechanism. In mature exposures, these reagents are external sulfates, calcium derived from CH dissolution and C-S-H decalcification, and aluminate derived from AFm dissolution. However, the major reagents used at an early age are external sulfates and aluminates derived from anhydrous cement and part of the AFm.

## 5. Conclusions

The aim of these experimental investigations was to better understand the kinetics of sulfate diffusion and its effects on the microstructure of ordinary cement paste. In addition, the ESA mechanisms were studied using various and complementary investigation techniques for both early-age and mature curing conditions. Based on the obtained results, conclusions can be drawn as follows:

- The sulfate profiles in the two curing cases are similar, with a slight difference in the maximum sulfate content. There is a more significant effect in the first three millimeters when the exposure occurs at an early age. During this early curing, the material is highly porous and permeable. Chemical interactions with the cement matrix delay sulfate ingress.
- The physical and chemical interactions with cement paste hydrates appear to have faster kinetics than diffusion through concentration gradients and capillary adsorption. This finding confirms that ESA is characterized by both the diffusion and binding of sulfate ions in the cement matrix, with mainly a chemical fixation.
- Matured Portland cement paste showed rapid degradation. This is due to the presence of a significant quantity of compressed ettringite and gypsum, as highlighted by SEM analysis. On the other hand, Portland cement pastes that were exposed to sulfate solution early in the process did not develop cracks or spalls after one year of exposure.
- Although AFt formation is known to cause the degradation of cementitious materials when they are exposed to sulfate ions, the chemical mechanism varies with curing duration. At an early age, the main reagents are external sulfates and aluminates from the anhydrous cement and part of AFm. In the mature exposition case, these reagents are external sulfates, calcium derived from the dissolution of CH and decalcification of C-S-H, and aluminates derived from the total dissolution of AFm.

## Figures and Tables

**Table 1:** Chemical composition of ordinary cement CEM I.

Chemical Composition (%)	CEM I
CaO	62.81
SiO_2_	20.22
Al_2_O_3_	4.85
Fe_2_O_3_	2.92
CaO (free)	1.58
MgO	0.84
SO_3_	2.88
S	0
K_2_O	0.77
Na_2_O	0.34
Ignition Loss	2.59

## Data Availability

All data are in this article. We don’t have other data to propose.

## References

[1] Zhao G., Shi M., Guo M., Fan H. (2020). Degradation Mechanism of Concrete Subjected to External Sulfate Attack: Comparison of Different Curing Conditions. Materials.

[2] Wagner M., Heisig A., Machner A., Beddoe R., Heinz D. (2022). External Sulfate Attack on Cementitious Binders: Limitations and Effects of Sample Geometry on the Quantification of Expansion Stress. Materials.

[3] Liu G., Tang Y., Wang J. (2023). Effects of carbonation degree of semi-dry carbonated converter steel slag on the performance of blended cement mortar—Reactivity, hydration, and strength. J. Build. Eng..

[4] Flatt R.J., Roussel N., Cheeseman C.R. (2012). Concrete: An eco-material that needs to be improved. J. Eur. Ceram. Soc..

[5] Planel D., Sercombe J., Le Bescop P., Adenot F., Torrenti J.-M. (2006). Long-term performance of cement paste during combined calcium leaching–sulfate attack: Kinetics and size effect. Cem. Concr. Res..

[6] Kaddah F., Ranaivomanana H., Amiri O., Rozière E. (2022). Accelerated carbonation of recycled concrete aggregates: Investigation on the microstructure and transport properties at cement paste and mortar scales. J. CO2 Util..

[7] Xu Z., Ye G. (2023). Understanding Chloride Diffusion Coefficient in Cementitious Materials. Materials.

[8] Martin R.-P., Omikrine-Metalssi O., Toutlemonde F. (2013). Importance of considering the coupling between transfer properties, alkali leaching and expansion in the modelling of concrete beams affected by Internal Swelling Reactions. Constr. Build. Mater..

[9] Omikrine-Metalssi O., Kchakech B., Lavaud S., Godart B. (2016). A new model for the analysis of the structural/mechanical performance of concrete structures affected by DEF—Case study of an existing viaduct. Struct. Concr..

[10] Al Shamaa M., Lavaud S., Divet L., Nahas G., Torrenti J.-M. (2015). Influence of relative humidity on delayed ettringite formation. Cem. Concr. Compos..

[11] Santhanam M., Cohen M.D., Olek J. (2003). Effects of gypsum formation on the performance of cement mortars during external sulfate attack. Cem. Concr. Res..

[12] Chen X., Gu X., Xia X., Li X., Zhang Q. (2021). A Chemical-Transport-Mechanics Numerical Model for Concrete under Sulfate Attack. Materials.

[13] El-Hachem R., Rozière E., Grondin F., Loukili A. (2012). Multi-criteria analysis of the mechanism of degradation of Portland cement based mortars exposed to external sulphate attack. Cem. Concr. Res..

[14] Neville A. (2004). The confused world of sulfate attack on concrete. Cem. Concr. Res..

[15] Chen W., Huang B., Yuan Y., Deng M. (2020). Deterioration Process of Concrete Exposed to Internal Sulfate Attack. Materials.

[16] Schmidt T., Lothenbach B., Romer M., Neuenschwander J., Scrivener K. (2009). Physical and microstructural aspects of sulfate attack on ordinary and limestone blended Portland cements. Cem. Concr. Res..

[17] Jabbour M., Quiertant M., Baroghel-Bouny V. (2022). A Critical Review of Existing Test-Methods for External Sulfate Attack. Materials.

[18] Santhanam M., Cohen M.D., Olek J. (2003). Mechanism of sulfate attack: A fresh look Part 2. Proposed mechanisms. Cem. Concr. Res..

[19] Wagner M., Decker M., Kunther W., Machner A., Beddoe R.E., Heisig A., Heinz D. (2023). Gypsum formation mechanisms and their contribution to crystallisation pressure in sulfate resistant hardened cement pastes during early external sulfate attack at low sulfate concentrations. Cem. Concr. Res..

[20] Yu C., Scrivener K. (2013). Mechanism of expansion of mortars immersed in sodium sulfate solution. Cem. Concr. Res..

[21] Zuo X.-B., Zheng Z.-K., Li X.-N., Zou Y.-X., Li L. (2023). Mesoscale numerical simulation on the deterioration of cement-based materials under external sulfate attack. Eng. Fail. Anal..

[22] Ran B., Omikrine-Metalssi O., Fen-Chong T., Dangla P., Li K. (2023). Pore crystallization and expansion of cement pastes in sulfate solutions with and without chlorides. Cem. Concr. Res..

[23] Ragoug R., Metalssi O.O., Barberon F., Torrenti J.-M., Roussel N., Divet L., de Lacaillerie E. (2019). Durability of cement pastes exposed to external sulfate attack and leaching: Physical and chemical aspects. Cem. Concr. Res..

[24] Metalssi O.O., Touhami R.R., Barberon F., de Lacaillerie E., Roussel N., Divet L., Torrenti J.-M. (2023). Understanding the degradation mechanisms of cement-based systems in combined chloride-sulfate attack. Cem. Concr. Res..

[25] Shao W., Li Q., Zhang W., Shi D., Li H. (2023). Numerical modeling of chloride diffusion in cement-based materials considering calcium leaching and external sulfate attack. Constr. Build. Mater..

[26] Xiong C., Jiang L., Xu Y., Chu H., Jin M., Zhang Y. (2016). Deterioration of pastes exposed to leaching, external sulfate attack and the dual actions. Constr. Build. Mater..

[27] Rozière E., Loukili A., El Hachem R., Grondin F. (2009). Durability of concrete exposed to leaching and external sulphate attacks. Cem. Concr. Res..

[28] Bary B., Leterrier N., Deville E., Le Bescop P. (2014). Coupled chemo-transport-mechanical modelling and numerical simulation of external sulfate attack in mortar. Cem. Concr. Compos..

[29] Wu Q., Ma Q., Huang X. (2021). Mechanical Properties and Damage Evolution of Concrete Materials Considering Sulfate Attack. Materials.

[30] Wang P., Mo R., Zhou X., Xu J., Jin Z., Zhao T. (2021). A chemo-thermo-damage-transport model for concrete subjected to combined chloride-sulfate attack considering the effect of calcium leaching. Constr. Build. Mater..

[31] Feng P., Garboczi E.J., Bullard C.M.J.W. (2015). Microstructural origins of cement paste degradation by external sulfate attack. Constr. Build. Mater..

[32] Biczok I. (1967). Concrete Corrosion Concrete Protection.

[33] Kanaan D., Soliman A.M., Suleiman A.R. (2022). Zero-Cement Concrete Resistance to External Sulfate Attack: A Critical Review and Future Needs. Sustainability.

[34] Brown P.W. (1981). An evaluation of sulfate resistance of cements in a controlled environment. Cem. Concr. Res..

[35] (2004). Standard Test Method for Length Change of Hydraulic-Cement Mortars Exposed to a Sulfate Solution.

[36] (2006). Standard Test Method for potential Expansion of Portland-Cement Mortars Exposed to Sulphate.

[37] (2009). Standard Specification for Portland Cement.

[38] Massiot D., King I., Calvé S.L., Alonso B., Durand J.O., Bujoli B., Gan Z., Hoatson G. (2002). Modelling one and two-dimensional solid-state NMR spectra, Magn. Reson. Chem..

[39] de Lacaillerie J.-B.D., Fretigny C., Massiot D. (2008). MAS NMR Spectra of Quadrupolar Nuclei in Disordered Solids: The Czjzek Model. J. Magn. Reson..

[40] Richardson I.G. (2014). Model structures for C-(A)-S-H(I), Acta Crystallographica Section B Structural Science. Cryst. Eng. Mater..

[41] Kunther W., Lothenbach B., Skibsted J. (2015). Influence of the Ca/Si ratio of the C-S-H phase on the interaction with sulfate ions and its impact on the Ettringite crystallization pressure. Cem. Concr. Res..

